# Comparison of Pedicle Coagulation Hemorrhoidectomy With LigaSure Versus Conventional Milligan Morgan Hemorrhoidectomy in Reducing Post-operative Pain: A Randomized Controlled Trial

**DOI:** 10.7759/cureus.45015

**Published:** 2023-09-11

**Authors:** Asad Amir, Aamna Nazir, Amjad Umair, Muhammad Atif Khan, Shahzaib Maqbool, Muhammad I Anwar, Faizan Fazal

**Affiliations:** 1 Department of Surgery, Holy Family Hospital, Rawalpindi, PAK; 2 Department of Surgery, Rawalpindi Medical University, Rawalpindi, PAK; 3 Department of Medicine, Rawalpindi Medical University, Rawalpindi, PAK

**Keywords:** randomised controlled trial, post-operative pain management, haemorrhoidectomy, milligan morgan, ligasure

## Abstract

Background

Hemorrhoids refer to the abnormal enlargement of the anal cushions. They are a common anorectal problem with a prevalence of 5% in the general population aged greater than 40 years. The objective of this study was to compare Milligan-Morgan open hemorrhoidectomy with pedicle ligation with LigaSure (Medtronic, Dublin, Ireland) in terms of postoperative pain on day 1 and day 7. It is important to assess the technique that is associated with lower postoperative pain because both of these techniques are still practiced in the developing world.

Methods

It was a randomized controlled trial conducted in the Department of Surgery, Rawalpindi, Pakistan. A total of 100 patients were selected and were allotted into the two groups by lottery method. Patients aged from 15 to 60 years who presented with symptomatic third and fourth-degree hemorrhoids were included after taking informed consent. Patients who had a previous or concomitant anorectal disease, patients who had undergone previous surgery for hemorrhoids, and those who were anesthetically unfit for surgery (American Society of Anesthesiologists (ASA) class 3 or above) were excluded from the study. Pain was assessed using the Visual Analogue scale (VAS). Data was entered and analyzed using SPSS v. 23.0 (IBM Corp., Armonk, USA). Chi-square tests were applied. P-value <0.05 was taken as statistically significant.

Results

Out of 100 patients, 68 (68%) were males while 32 (32%) were females. The mean age was 40.56±9.24 years. Postoperative pain at day 1 was 9.24±0.51 in the Milligan-Morgan group while that in the LigaSure group was 8.44±0.64 (p<0.0001). Postoperative pain at day 7 was 5.00±0.85 in the Milligan-Morgan group while it was 3.04±1.08 in the LigaSure group (p<0.0001).

Conclusion

LigaSure is a newer technique that helps to reduce complications as compared to other traditional hemorrhoidectomy procedures. Many patients avoid hemorrhoidectomy as it is associated with painful postoperative recovery. Pedicle coagulation with LigaSure was better than conventional Milligan-Morgan hemorrhoidectomy in terms of reducing the mean postoperative pain on 1st day and 7th day. Reducing the postoperative pain helps in greater patient satisfaction and lesser requirement of analgesia among patients of 3rd and 4th-degree hemorrhoids undergoing hemorrhoidectomy.

## Introduction

Hemorrhoids are normally found in the human body [1-3]. Internal hemorrhoidal plexus gives rise to internal hemorrhoids whereas external hemorrhoidal plexus gives rise to external hemorrhoids. Anatomically, the internal hemorrhoidal plexus is separated from the external one by the dentate line [1,2,4]. Normally, the internal hemorrhoidal plexus consists of three soft vascular cushions known as anal cushions or “hemorrhoids” [5,6]. Thus, the term “internal hemorrhoids” is not a disease according to the anatomical definition but clinically, this term means the abnormal enlargement of these anal cushions [2,5,6]. Hemorrhoids are a common anorectal problem with a prevalence of 5% in the general population aged greater than 40 years [7]. Hemorrhoids are the fourth leading outpatient gastrointestinal diagnosis, accounting for approximately 3.3 million ambulatory care visits in the United States [8].

Symptoms of hemorrhoids include bright red painless per rectal bleed, mucus discharge, and prolapsed anal mucosa. If left untreated, they may lead to complications like strangulation, thrombosis, ulceration, gangrene, fibrosis, or portal pyemia [9]. A hemorrhoidectomy is the standard treatment for patients with grade III or IV internal hemorrhoids [10]. Milligan-Morgan open hemorrhoidectomy remains a very popular treatment modality for third and fourth-degree hemorrhoids due to its cost-effectiveness and good long-term results. The complications include blood loss leading to prolonged operative time, postoperative pain leading to a prolonged hospital stay, a greater requirement for analgesics, and delayed return to work or daily activities [9]. With recent advances, hemorrhoidectomies are now being performed with new devices, such as bipolar electro-thermal devices, ultrasonic scalpels, and circular staplers.

The LigaSure (Medtronic, Dublin, Ireland) is a novel device consisting of a bipolar electro-thermal device with an optimized combination of pressure and radiofrequency, with the ability to seal blood vessels up to 7mm in diameter and associated with a collateral thermal injury confined to 2mm over the surgical field. This confined spread decreases reflex anal spasm and a bloodless hemorrhoidectomy is possible with reduced postoperative pain and rapid healing. Thus, this operation can be advocated as the ideal technique because of the potentially decreased collateral tissue trauma [11]. Among the postoperative complications, many patients experience postoperative pain after undergoing a hemorrhoidectomy and many patients complain of discomfort for a long time [12]. The efficacy of hemorrhoidectomy by LigaSure is better than the traditional Milligan-Morgan hemorrhoidectomy but we need more clinical trials with large sample sizes and long-term follow-ups to show that LigaSure is better than Milligan-Morgan hemorrhoidectomy [11].

The aim of this study is to evaluate the LigaSure pedicle coagulation in comparison with conventional Milligan-Morgan hemorrhoidectomy pedicle ligation and if proven effective it will result in less postoperative pain, more patient satisfaction, and less requirement of analgesia.

## Materials and methods

Study setting and duration

This study was a randomized controlled trial conducted at the Department of Surgery Holy Family Hospital, Rawalpindi from 10th October 2021 to 10th April 2022.

Ethical approval

This randomized controlled trial was approved by the Ethical Review Board of Rawalpindi Medical University. The reference number is SUR-96-46-23.

Randomization

A total of 100 patients were divided into two groups of 50 each and were randomly allotted into one of the two groups by lottery method. Group A comprised of patients undergoing Milligan-Morgan open hemorrhoidectomy and group B comprised of patients who underwent pedicle coagulation by LigaSure. Randomization was single-blinded meaning that the patient did not know about the one procedure that was going to be used among the two procedures.

Eligibility criteria

Both male and female patients aged 15 to 60 years, who presented with symptomatic third, and fourth-degree hemorrhoids were included after taking informed consent. Patients who had a previous or concomitant anorectal disease, patients who had undergone previous surgery for hemorrhoids, and those who were anaesthetically unfit for surgery (ASA class 3 or above) were excluded from the study.

The procedure of pedicle coagulation hemorrhoidectomy

After administering standardized spinal or general anesthesia the procedure was carried out with the patient in the lithotomy position. After examination under anesthesia, hemorrhoids were delivered with the help of artery forceps. One of the artery forceps was applied at the mucocutaneous junction of the hemorrhoid and the other at the apex of the hemorrhoidal tissue. A skin incision was given at the base of the hemorrhoids and dissection was done to separate the hemorrhoidal tissue from the internal anal sphincters. After this, in Milligan-Morgan’s open technique, the hemorrhoid pedicle was transfixed with zero number Vicryl suture while in the other group, the hemorrhoid pedicle was coagulated with the help of LigaSure.

Postoperative procedure

**Figure 1:**
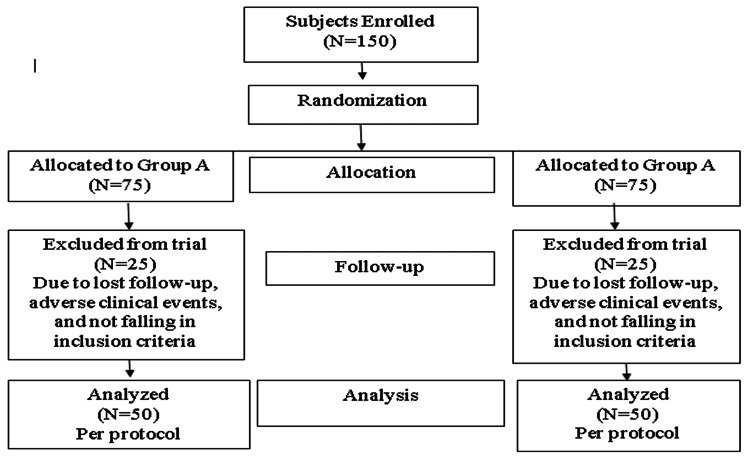
Showing the CONSORT flow chart of study selection CONSORT: Consolidated Standards of Reporting Trials

Postoperatively patient was given a ketorolac injection of 30 mg 8 hourly and pain was recorded on a visual analog pain score ranging from 0 to 10 after 24 hours. Follow-up was done on the seventh postoperative day in the surgical outpatient department. Mean post-operative pain was calculated. This randomized controlled trial was done in accordance with Consolidated Standards of Reporting Trials (CONSORT) guidelines as shown in Figure 1 [13].

Data analysis

150 subjects were initially enrolled in the study. 50 patients were excluded and a total of 100 patients were divided into two groups of 50 each. The collected data was entered in the SPSS Statistics v. 20.0 (IBM Corp., Armonk, USA). Qualitative variables like gender, ASA grade, and grade of hemorrhoids were measured as frequencies and percentages. The quantitative variables like age and postoperative pain were measured as mean ± SD. Independent samples t-test was used to compare postoperative pain in both groups. Effect modifiers like age, gender ASA class, and grade of hemorrhoids were controlled by stratification. Post-stratification chi-square test was applied. P-value ≤ 0.05 was significant.

## Results

A total of 100 patients were included in the study out of which 68 (68%) were males whereas 32 (32%) were females. Both genders were equally distributed within the study groups. There were 34 males and 16 females in each of the two groups. The mean age of the study participants was 40.56±9.24 years. The mean age of the patients in group A (Milligan-Morgan open hemorrhoidectomy) was 43.84±9.98 years and that in group B (pedicle coagulation by LigaSure) was 37.28±7.15 years. Out of 100 patients, 82 (82%) belonged to ASA class I while the rest 18 (18%) were of ASA class II. Thirty-six out of 82 (43.9%) in ASA class I and 14/18 (77.78%) in class II were in study group A. Forty-six out of 82 (56.1%) in class I and 4/18 (22.22%) in class II were in study group B.

**Figure 2:**
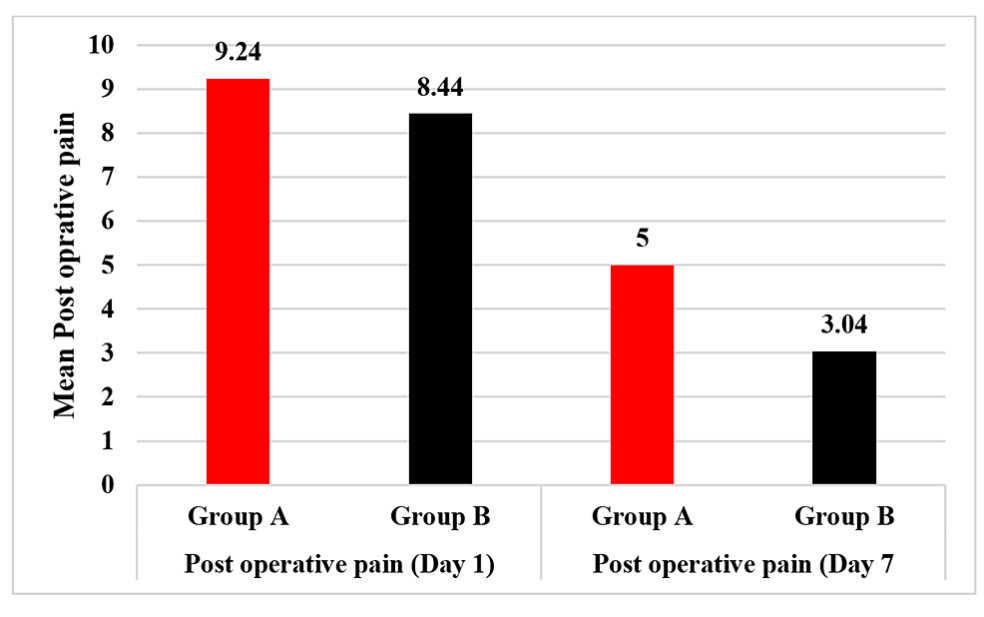
Comparison of mean postoperative pain at day 1 and day 7 in the patients undergoing Milligan-Morgan Open Hemorrhoidectomy (Group A) and pedicle coagulation by LigaSure (Group B)

Eighty-four out of 100 (84%) patients had grade 3 hemorrhoids while 16/100 (16%) had grade 4 hemorrhoids. Out of 84 patients with grade 3 hemorrhoids, 38 (45.24%) were in study group A while 46 (54.76%) were in study group B. Similarly, out of 16 patients with grade 4 hemorrhoids 12 (75%) were in group A while 4 (25%) were in group B. postoperative pain on day 1 was 9.24±0.51 in the Milligan-Morgan group while that in the LigaSure group was 8.44±0.64. This difference in mean postoperative pain at day 1 was statistically significant between the two groups (p<0.0001). postoperative pain on day 7 was 5.00±0.85 in the Milligan-Morgan group while it was 3.04±1.08 in the LigaSure group. Mean postoperative pain at day 7 was also statistically significant between the two groups (p<0.0001). postoperative pain on both day 1 and day 7 was significantly lesser in the LigaSure group which means that the LigaSure procedure is associated with better postoperative clinical outcomes as compared to the Milligan-Morgan procedure. A comparison of mean postoperative pain in both study groups is shown in Figure 2.

The results of stratification for postoperative pain at day 1 and day 7 with regards to age, gender, ASA class, and hemorrhoids grades are shown in Table 1.

**Table 1:** Stratification for postoperative pain at day 1 and day 7 with regards to age, gender, ASA class, and hemorrhoids grades ASA: American Society of Anesthesiologists

Dependent variables (Outcome variables)	Independent variables (Explanatory variables)	Groups	Post-operative pain in Milligan Morgan Open Hemorrhoidectomy (mean ± S.D)	Post-operative pain in Pedicle Coagulation by LigaSure (mean ± S.D)	P value
Postoperative pain on Day 1	Age	15 – 25 years	8.00+0.00	8.12+0.53	0.014
		26 - 60 years	8.88+0.00	8.08+0.88	
Postoperative pain on Day 7		15 – 25 years	2.00+0.00	6.26+0.19	0.002
		26 - 60 years	4.10+1.34	7.21+0.31	
Postoperative pain on Day 1	Gender	Male	8.85+0.94	8.12+0.44	0.791
		Female	8.81+0.09	8.01+0.18	
Postoperative pain on Day 7		Male	4.06+1.42	4.35+0.86	0.685
		Female	3.94+1.31	3.91+0.81	
Postoperative pain on Day 1	ASA Grade	Grade I	8.78+0.68	8.12+0.61	0.072
		Grade II	9.11+0.75	9.06+0.08	
Postoperative pain on Day 7		Grade I	3.85+1.43	5.26+0.03	0.01
		Grade II	4.78+0.80	6.12+0.44	
Postoperative pain on Day 1	Hemorrhoids Grade	Grade 3	8.76+0.68	7.12+0.21	0.011
		Grade 4	9.25+0.68	6.02+0.15	
Postoperative pain on Day 7		Grade 3	3.93+1.32	4.16+0.03	0.131
		Grade 4	4.50+1.63	4.51+1.64	

## Discussion

Hemorrhoidectomy is an effective treatment for 3rd and 4th-degree hemorrhoids [14]. However, many patients avoid hemorrhoidectomy as it is associated with painful postoperative recovery [11]. Many other procedures are also used including laser therapy, stapled hemorrhoidectomy, open or closed sharp excision, and ultrasonic scalpel dissection [15-18]. LigaSure is a newer technique that helps to reduce complications as compared to other traditional hemorrhoidectomy procedures [19,20]. In our study, hemorrhoids were more common in males (68%) as compared to females (32%). This is similar to the results shown by Ravindranath and Rahul, where 66.67% were males while 33.33% were females [21]. In a study by Ali and Shoeb, 55% of males had hemorrhoids as compared to 45% of females [22].

In our study, the mean age of the patients with hemorrhoids was 40.56 years. This is consistent with the study by Ravindranath and Rahul, where the mean age was below 40 years of age [21]. Ali and Shoeb showed that the most common age group who presented with hemorrhoids was 20-39 years [22]. In contrast to this, Khan et al. showed that patients aged above 40 years were more at risk as compared to those below 40 years [23]. In our study mean score of postoperative pain on day 1 was 9.24 in the open hemorrhoidectomy group as compared to the mean pain score of 8.44 in pedicle ligation with the LigaSure group. Bakhtiar et al. also reported a higher pain score in the Milligan-Morgan group (5.41) as compared to the LigaSure group (3.65) [11]. On day 7, the mean pain score was five in the Milligan-Morgan group as compared to the mean score of 3.04 in the LigaSure group in our study. Similarly, in the study by Bakhtiar et al., the mean pain score on the 7th postoperative day was higher in the Milligan-Morgan group (2.44) as compared to the LigaSure group (1.34) [11]. This shows that LigaSure is a relatively pain-free technique as compared to Milligan-Morgan's open hemorrhoidectomy. Furthermore, in the Milligan-Morgan technique, the hemorrhoid pedicle is ligated by a transfixing suture. This suture leads to postoperative pain, wound infection, and bleeding. These postoperative complications are not seen in the Ligasure technique because no sutures are used in this technique. This might be one of the rationale behind less postoperative pain seen among the LigaSure group [11].

The results of stratification showed that postoperative pain at day 1 was higher in the age group 26-60 years (8.88) as compared to the age group 15-25 years (8.00) in the Milligan-Morgan group. Similarly, postoperative pain at day 7 was also higher in the age group 26-60 years as compared to 15-25 years in both the study groups Postoperative pain at day 1 was higher in grade 4 hemorrhoids (9.25) as compared to grade 3 hemorrhoids (8.76) in the Milligan-Morgan group. Whereas postoperative pain at day 7 was more in grade 3 (7.12) as compared to grade 4 hemorrhoids (6.02) in the LigaSure group. 

## Conclusions

LigaSure is a newer technique that helps to reduce complications as compared to other traditional hemorrhoidectomy procedures. Many patients avoid hemorrhoidectomy as it is associated with painful postoperative recovery. This study concluded that pedicle coagulation with LigaSure was better than conventional Milligan-Morgan hemorrhoidectomy in terms of reducing the mean postoperative pain on 1st day and 7th day. Reducing the postoperative pain helps in greater patient satisfaction and lesser requirement of analgesia among patients of 3rd and 4th-degree hemorrhoids undergoing hemorrhoidectomy.
